# End the AI detection arms race

**DOI:** 10.1016/j.patter.2024.101058

**Published:** 2024-10-11

**Authors:** J. Scott Christianson

**Affiliations:** 1 University of Missouri, Trulaske College of Business, Columbia, MO, USA

## Abstract

The advent of easy-to-use large language models (LLMs) such as ChatGPT has started an arms race in academia between students who use AI and faculty trying to detect that use. This unproductive battle must end, and faculty can help broker peace by rethinking assignments and using LLMs where appropriate.

## Main text

While large language models (LLMs), such as ChatGPT, have numerous flaws, they are excellent “word calculators” that can easily mimic human use of language and understanding of our world. No group in education was quicker to recognize the potential of LLMs than students, who quickly put the essay writing, question answering, and tutoring abilities of LLMs to use after the November 2022 release of ChatGPT by OpenAI. Less than 20 days after ChatGPT’s public availability, some Stanford students used it to help with their Fall quarter final exams.^1^ Seventeen percent of the 4,497 Stanford students informally surveyed reported using ChatGPT for their exams, and five percent of those used output from ChatGPT without any edits. Rapid adoption at Stanford was not surprising—OpenAI’s leader Sam Altman is a Stanford dropout—but adoption spread like wildfire worldwide, quickly making ChatGPT the fastest-adopted platform in history.^2^

A discussion immediately started about how AI-generated materials could be detected and flagged. While AI-generated images can be watermarked, either explicitly or in ways that hide the watermark, text generated by AI can’t be easily tagged as being AI-generated, and if it is tagged, those tags can be easily removed.

Several companies rapidly deployed tools that purported to recognize patterns indicative of AI writing. Initially, this technology looked promising, and even OpenAI released its own AI detector in early 2023. An early startup developed by a Princeton grad, GPTZero, analyzed text in terms of burstiness (how repetitive or clustered a group of words is) and perplexity (how predictable the word use is) and became very popular.

However, people soon discovered that the generating prompts could be altered or the output text could be edited to avoid detection.^3^ Numerous startups launched to help students make AI-generated text undetectable. The AI generation vs. AI detection arms race began to escalate, and Turnitin and other established Ed-Tech companies became the arms dealers for faculty headed into battle.

Unfortunately, many faculty misused AI-detection tools during those early days, leading to much “collateral damage” and headlines about professors failing entire classes because the professor did not understand how to use AI detection.^4^ Hundreds of TikToks showed the anguish of accused students, encouraging the public to adjudicate their case.

To complicate matters further, it quickly became apparent that AI detectors had high false positives and negatives.^5^ These systems also discriminated against non-native English speakers.^6^ OpenAI shut down its detector relatively swiftly and warned that such systems were unreliable.^7^ GPTZero pivoted to producing AI writing tools that instructors can use to monitor students as they write. Yet, that didn’t stop other Ed-Tech companies from baking AI detection tools into their systems and selling them to institutions at all levels.

While faculty and administrators searched for new tools to help them maintain the status quo, most students saw clearly what these new tools revealed about higher education.

Writing in *The Princetonian* in February 2023, student Christopher Lidard concluded,

… if students are being assigned essays that can be written by ChatGPT, perhaps it’s not a good assignment in the first place. ChatGPT begs us to rethink the purpose and value of homework. Some would argue that ChatGPT subverts the purpose of a good education by providing students with instant, personalized assistance on a wide range of subjects and topics. Yet if Princeton homework can be completed by a machine, what is its true value?^8^

The type of student assignments Lidard refers to are a form of *a priori* knowledge on the faculty’s part. We know what the student’s response should be before the student goes through the research and writing experience. For example, suppose I assign a student to write an essay about Harry S. Truman’s significant achievements during his presidency. In that case, I know what should be in that student’s response: something about FDR’s death, dropping the bomb, integrating the military, firing McArthur, recognizing Israel, etc.

This type of assignment works well in our Taylorist factory model of undergraduate education, with large classes and armies of teaching assistants trained to process papers. And I hate to say it, but some faculty also use AI-based grading programs to provide initial scoring for these types of assignments.

Of course, these are the types of essays that are super simple for an LLM such as ChatGPT to write and for essay-writing companies to churn out in the pre-ChatGPT days, lest we forget that cheating didn’t start with LLMs.

A better assignment is one where the faculty and student don’t know the answer in advance; the knowledge comes *a posteriori* or after the student’s experience. For example, what was the impact of President Truman’s decision on the trajectory of the student’s family or the place where they grew up? I don’t know the answer to that, and neither does the student or ChatGPT. An LLM could help the student write up their research but can’t do it well on its own. This example is also a much more interesting type of assignment for the student and faculty member: one that creates knowledge.

While epistemologists might have issues with my use of *a priori* and *a posteriori*, I find these valuable constructs when designing assignments. ChatGPT forces us to discard the use of homework assignments as a tool for evaluation and turn it into an opportunity to expand students’ understanding of core concepts through original research and insight. It also allows us to raise our expectations of what we expect from students, especially if we allow some use of LLMs in key areas of our assignments.

Showing your students how to use an LLM for their assignments or co-learning it with them offers an opportunity to de-escalate the situation and restore trust in the faculty-student relationship. Even more rewarding is that incorporating LLM use in your class is a gateway to teaching how to ask good questions in your field, as getting good results from LLMs requires asking good questions.

However, *a posteriori* project-based assignments are not amenable to the industrial production of education. ChatGPT will not destroy education, but I am hopeful it will finally blow up the large lecture and huge student credit load many faculty have to bear.

Unfortunately, in many institutions this Fall, the AI detection arms race will continue, pitting institutional goals (massive classes and easily gradable assignments) against student goals (clearing mundane assignment hurdles so they can move on to more significant challenges). It is a race that can’t be won. To borrow a line from the movie *Wargames*, the only winning move in the AI-Writing vs. AI-Detection war is not to play.

## Declaration of interests

The author declares no competing interests.
